# A Rare Case of Traumatic Cervical Spinal Epidural Hematoma in a Toddler: A Two-Year Follow-Up

**DOI:** 10.7759/cureus.31767

**Published:** 2022-11-21

**Authors:** 'Afif Abdul Latiff, Mohd Hisam Muhamad Ariffin

**Affiliations:** 1 Orthopaedics and Traumatology, Universiti Kebangsaan Malaysia Medical Centre, Kuala Lumpur, MYS; 2 Spine Surgery, Universiti Kebangsaan Malaysia Medical Centre, Kuala Lumpur, MYS

**Keywords:** traumatic spinal epidural hematoma, traumatic pediatric quadriplegia, cervical spine injury, traumatic spinal hematoma, cervical epidural hematoma

## Abstract

Traumatic cervical epidural hematoma is a rare disease in the pediatric population. It requires a high level of suspicion in children who presents with acute neurological deficit after trauma. Magnetic resonance imaging (MRI) is required to confirm the diagnosis. Early surgical intervention is recommended to have the best neurological outcome. We report a case of a traumatic cervical epidural hematoma in a toddler with complete paraplegia, which partially recovered after decompressive surgery. We would like to emphasize the importance of high suspicion for this condition and the need for an urgent MRI to confirm the diagnosis.

## Introduction

Jackson first described spinal epidural hematoma (SEH) in 1869, which accounts for less than 1% of all space-occupying lesions of the spinal canal [1]. It is an important topic as delaying the diagnosis and treatment is harmful as it is a disabling disease. Based on the literature, the estimated incidence per year is 0.1 out of 100,000 per year [1,2]. This pathology can be due to trauma or can be spontaneous. Post-traumatic epidural hematomas are rare in comparison to spontaneous hematomas, which are more common [2]. We share a case of a toddler who had a traumatic cervical epidural hematoma with quadriplegia, which was treated with decompressive surgery with a good recovery.

## Case presentation

A 22-month-old toddler with no known medical illness presented to us with a history of a fall five days prior to her presentation to our center. Her developmental milestones were up to age prior to the trauma. The child fell on the ground with a hyperextended neck from about 1-m height. She did not sustain any loss of consciousness and was still able to run and play as usual immediately post-fall. One day after the trauma, she started to gradually lose her motor power and was unable to move her upper and lower extremities. She also started to have bowel and urinary incontinence.

**Figure 1:**
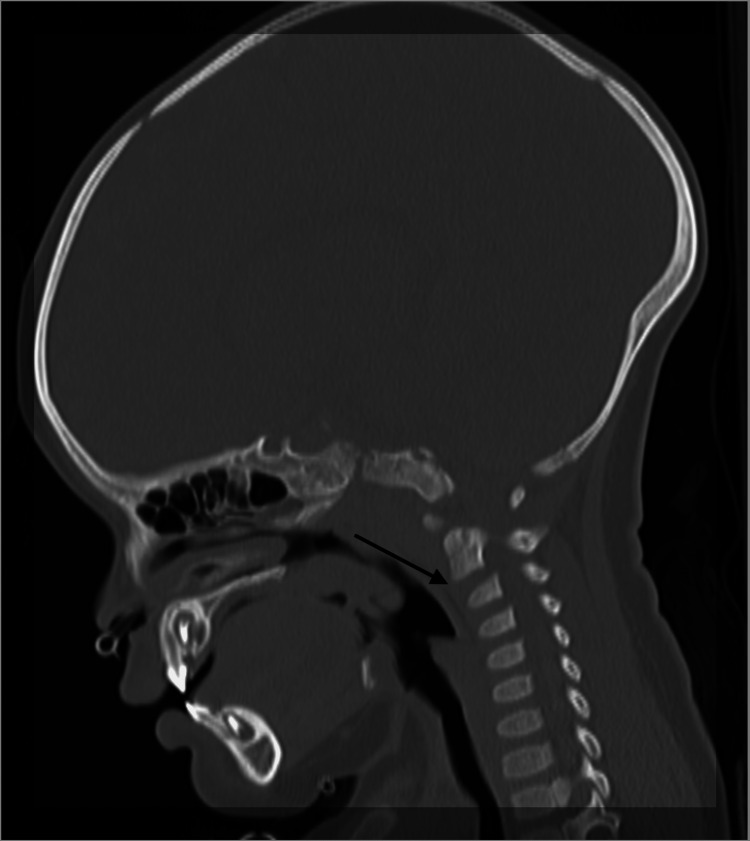
Sagittal cut section of the head and neck CT scan showing mild listhesis at the C2/C3 level. CT: computed tomography

**Figure 2:**
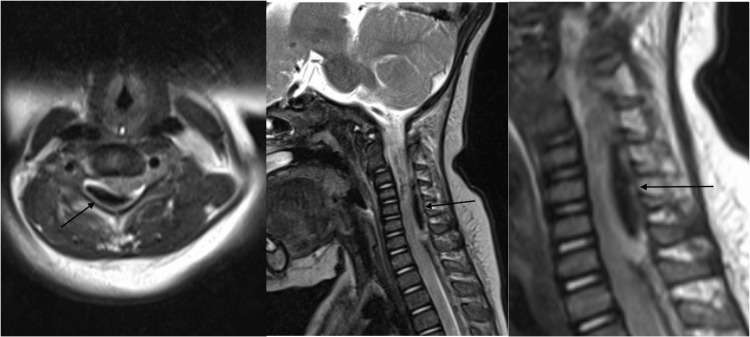
MRI T2-weighted image of axial and sagittal views showing a posterior hematoma from the C2 to T8 level predominantly at the C4 to T1 level causing canal stenosis. MRI: magnetic resonance imaging

During the initial presentation to the emergency department, her head and neck control was normal. She had hypotonia over all four limbs. Her upper and lower limb reflexes were absent. There was only flickering of muscle over bilateral upper and lower limbs. Computed tomography (CT) scans of the brain and neck were performed and showed no intracranial bleed; however, mild anterior displacement of C2 over C3 was revealed as shown in Figure 1. Magnetic resonance imaging (MRI) of the spine showed an acute right posterior cervical and thoracic epidural hematoma from the C2 to T8 level, predominantly over the C4 to T1 level. The hematoma was causing spinal cord compression with mild spinal cord edema at the C4 level as shown in Figure 2.

**Figure 3:**
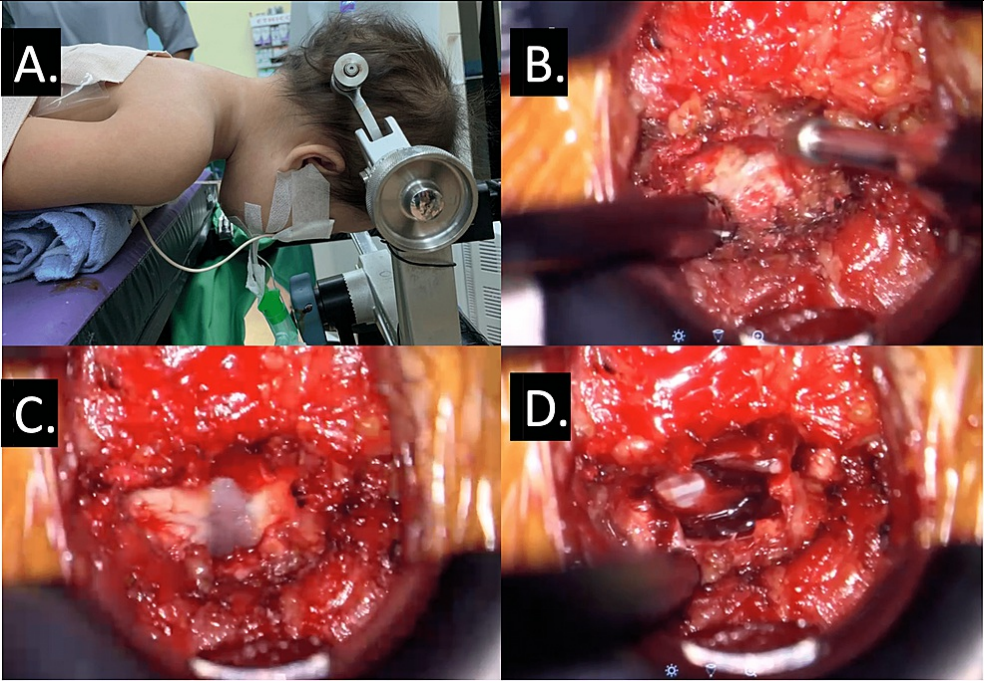
(A) The patient positioned on a spinal Jackson table with a Mayfield clamp attached to control cervical movement during positioning. (B) Laminectomy done at the most compressed level, which was C5. (C) Thick periosteum posterior of C5 overlying the hematoma and the spinal cord. (D) Intraoperative picture showing the hematoma at the peripheries surrounding the spinal cord at the C5 level, which was taken using a Zeiss exoscope.

C5 cervical decompressive surgery was planned immediately. The patient was positioned on a spinal Jackson table with a Mayfield clamp attached as shown in Figure 3. Laminotomy was done using a burr at the C5 level as shown in Video 1. There was a thick periosteum at the C5 level overlying the hematoma and the spinal cord as shown in Figure 3 and Video 2. Intraoperatively, it was noted that there was hematoma formation worst at the C5 region causing compression to the spinal cord as shown in Figure 3. Direct decompression was done by removing the hematoma as shown in Video 3. The spinal cord was pulsating, and there was return of epidural microcirculation after decompression.

**Video 1:** Laminotomy done at the level that was most compressed based on the MRI. MRI: magnetic resonance imaging

**Video 2:** Thick periosteum overlying the spinal canal.

**Video 3:** Removal of the hematoma that was causing the compression over the spinal cord.

The child showed significant neurological improvement postoperatively evidenced by the return of her left upper and lower limb motor power. However, she had residual weakness over her right upper and lower limbs. Other residual neurological deficits were neurogenic bladder and bilateral diaphragmatic paralysis.

**Figure 4:**
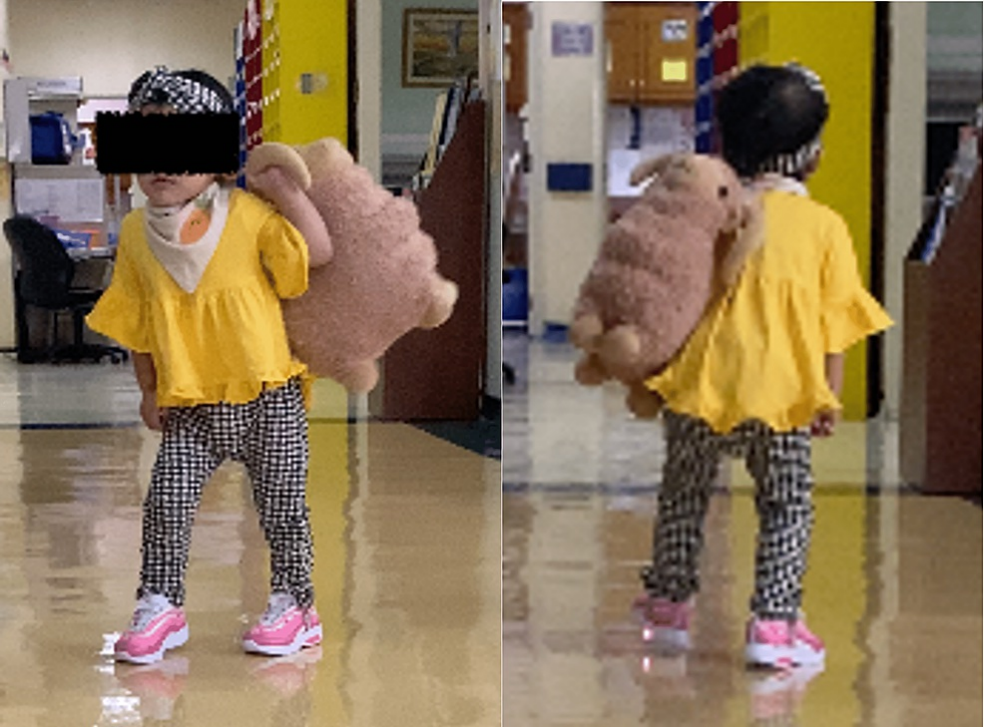
Clinical picture of the patient at two-year follow-up.

Upon her two-year follow-up after the surgery, she was able to stand, walk, and run unsupported. The motor power of her left upper and lower limbs was normal. Over her right side, she was able to extend her wrist with a strong grip power demonstrated by her ability to hold toys with her right hand. Her locomotor and self-care were a bit delayed. However, her cognitive, visual, hearing, and language developments were normal. Her clinical pictures during her follow-up are shown in Figure 4.

## Discussion

Spinal epidural hematoma (SEH) in pediatrics is a rare condition that can be either spontaneous or traumatic [1-4]. SEH represents less than 1% of space-occupying lesions of the spinal canal [2]. It is reported to represent 0.1 per 100,000 patients in the general population, and the occurrence in the pediatric population is much lower. The majority of cases are spontaneous, and traumatic spinal epidural hematomas are extremely rare. Based on a literature search done by authors, as of today, there were around 40 cases in total, and eight of them were traumatic [1,3].

A spontaneous epidural hematoma can be due to hematologic problems such as coagulopathies, liver diseases, infections, hematologic cancers, and anticoagulants. In cases of high-impact trauma, the development of SEH is related to the rotatory/whiplash mechanism of injury at the lower cervical region [5].

Children will usually present with nonspecific symptoms, which makes it difficult to diagnose [3]. Patients will generally present with symptoms that are caused by spinal cord injury, which include weakness such as hemiparesis, paraparesis, quadriparesis, paraplegia, and even quadriplegia and inability to control passing of motion or urination. Other symptoms would be neck pain, stiffness, crying, irritability, and a decrease in the range of motion of the neck [1].

Physicians should have a high level of suspicion of spinal pathology, and the gold standard for the diagnosis of SEH is to perform an MRI [2]. The MRI is then verified to locate the spinal epidural hematoma at the cervical region as found in our case. Another modality that can be used is CT angiography to supplement the MRI [1,3]. There are controversies regarding the source of bleeding whether it is coming from the artery or venous injury [5].

It was reported that the average days prior to surgery was 7.1 days (ranging from two to 14 days). Like in our case, the patient presented on day 5 as it was in the range of the other cases that have been reported, and she was operated on the same day. A few papers published advocate rapid surgical evacuation as the treatment of choice for patients with spinal epidural hematoma who present with symptoms [1,3,5]. Patients who underwent surgery within less than 24 hours of symptoms have the most favorable outcome. The surgical treatment of choice is typically hemilaminectomy or a laminectomy followed by debridement and irrigation [2,5]. However, nonsurgical treatment is an option for patients who present with no neurological deficit. There are reports showing regression and spontaneous relief of symptoms in these patients [5]. Delaying the diagnosis and treatment may lead to devastating and permanent neurological deficits from paresis down to even death [2].

## Conclusions

Traumatic cervical injuries with spinal epidural hematoma are relatively rare among the pediatric population. Patients usually present with nonspecific symptoms, and it should be high on the list of suspicion. MRI is the gold standard for diagnosing spinal epidural hematoma. Early diagnosis and surgical treatment have the most favorable outcome with regard to neurological improvement.
